# The Bromides

**Published:** 1870-01

**Authors:** F. K. Bailey

**Affiliations:** Knoxville, Tennessee


### ARTICLE III.

### THE BROMIDES.

BY F. K. BAILEY, M.D., Knoxville, Tennessee.

In prefacing a few observations upon the bromides, it may not be amiss to allude to bromine as a therapeutical agent.

It was extensively used as a topical application in hospital gangrene, during the war. In 1863 and 1864, a good many cases came under my care and observation, and nothing could be more obvious than its value in arresting the spread of the morbid influence, and placing the parts in a condition for healing. Pure and undiluted, it did not fail in a single instance, and many an ex-soldier can now attest its virtues, and will also say, that like the rod in the hands of a parent, it caused a smarting "while it made him better."

Of the bromide of sodium I have seen but little in its action upon the animal economy. By some it is more highly esteemed than the bromide of potassium.

The bromide of ammonium I have used somewhat in the last year, and found it very useful in some forms of nervous disturbances. It acts more directly upon the nervous system, and is valuable when stimulation is required. In my practice, during the last two or three years, I have used bromide of potassium very freely. Among the first cases I treated after coming to this city, was that of a lady aged about 35, married, and mother of one child, which died some years ago. I saw her in November, 1867, and found that during the spring previous she had an attack of acute cystitis. After the violence of the symptoms subsided, dysuria followed, continuing all summer, together with facial neuralgia. When I first saw her she was suffering from chronic inflammation of the mucous coat of the bladder. The urine was alkaline, and was loaded with a thick mucus, which filled sometimes one-half of a vial in which it had been collected. There was severe pain referred to the fundus, besides great irritation along the urethral canal, and at the external meatus. There was also a good deal of pain in the region of the kidneys, caused by sympathy, as well as some local congestion. There was emaciation; some cough, and great depression of spirits. There was a dark, yellowish hue to the skin, and troublesome constipation. Her former medical attendant, who was leaving the city, informed me that he had a short time before commenced the use of bromide potassium, with tonics and mild alteratives. I prescribed as follows:—

| | | | |
|---|---|---|---|
| ℞. | Bromide of potass., | | ʒiij. |
| | Fluid ext. stillingia,<br>Syr. aurantii cort., | } āā | ℥ss. |
| | Sulph. Cinchoniæ, | | ℈j. |
| | Aqua Cinnamonii,<br>Aqua Pura, | } āā | ℥j. |

M. F. Mix. Sig. Teaspoonful morning and night.
To take spts. nit. dulc. *pro re nata.*

The above was taken for several weeks, with the alternation at times, of syrup of iodide of iron. During the winter there began to be some improvement. But it was nearly a year before the mucus ceased to be voided. At times, when the urine was most alkaline, the mucus would be most abundant, and the distress in the bladder and dysuria would be aggravated. During the summer of 1868, she became quite comfortable, and took little or no medicine. In the autumn, there were some indications of a relapse, but a return to the bromide, with tonics, soon averted the disease. During the past year she has been scarcely at all troubled, and considers herself cured of cystitis.

During the autumn of 1867, I met with a case of epilepsy, which seemed a favorable one for the trial of the bromide. It was a colored man, of 50 years or more, and possessing more than the ordinary amount of intelligence for one of his race.

He purchased his freedom, and that of his wife, some years before the war, and worked on his own account as a tinner. Up to 1861, he had acquired some property, which, with his prospects for the future, soon vanished as the war began. Poverty and disappointment, consequent upon his reverses, caused a depression of spirits. Being something of a preacher as well as mechanic, in 1865 he began to be particularly interested in measures for the good of his fellow blacks. Mental excitement soon began to increase a former tendency to cerebral disturbance, which culminated in epilepsy, and in the summer of 1867 he had the first fit. He had taken bromide potass. for a short time before I saw him, and I continued its use, together with tonics and laxatives. For a year or more, the disease seemed stayed; but since last spring the fits have come on again, at long intervals, with the lesser symptoms intervening.

In the August number of the *Examiner*, are some cases reported by Salvator Cass, M.D., upon the use of this salt in summer complaint, and I prescribed it for a patient then under my care. It was a female child, 10 months old, that had suffered from difficult dentition for some weeks. Gave one and a-quarter grain, in mucilage, every four hours. Next day called, and found no diarrhœa, and that the child had slept well all night. Scarified the gums, and discharged the little patient as convalescing. I was told that the diarrhœa returned in about a week, but was promptly stopped by a resort to the syrup.

*September 5.* Called to see a female infant, 10 months old, that had been sick with cholera infantum about eight weeks. The little creature was emaciated, with profuse evacuations every few minutes, attended with nausea and vomiting. The tongue and mucous lining of the mouth were deep red, with intense thirst, and a burning heat of the skin. It had suffered long from tenesmus, and tormina, which were only relieved, as I was told, by injections of tinct. opii. Five or six teeth had already appeared, and more still coming. I prescribed bromide potassii at once, in one and a-quarter grain doses, with syr. aurantii, four times daily, alternated with four drops liq. iodidi ferri. Having been nursed from birth "upon a bottle," I directed undiluted milk, without sugar, as the diet, and to offer it water every time before putting the milk to its mouth. In a few days could be seen a very decided improvement. The diarrhœa and vomiting ceased, and the child could sleep quietly. For the last month she has become quite full in the face, and a few days ago it was necessary to adopt measures to obviate a tendency to constipation.

*September 28.* Called to see a male infant, 8 months old, that had been sick a month with diarrhœa, and vomiting. It was emaciated, and retained nothing upon the stomach. Its former medical attendant had told the mother that her child must die, and appearances certainly justified such a prognosis. I gave bromide potassii, as in the other cases, with an effect equally apparent. Prescribed, also, iod. iron, brandy punch, and quinine. For the last fortnight has required no medicine, and is doing well.

Another case was that of a female infant, 13 months old. Had diarrhœa in June last, which was arrested by means of calomel and ipecac, followed with quinia. October 7th, it was again taken, with considerable violence, on cutting the first molar teeth. The bromide was given at once, and the disease was controlled. Nothing more was done but to scarify the gums.

In the foregoing cases, not only was the diarrhœa suspended, but there was decided relief to pain, and the little patients would sleep quietly, as though under the influence of opium. I have, also, in a few cases, prescribed the bromide for children who were wakeful and fretful at night, with decidedly good effects.

In one or two cases, I have prescribed the bromide, with fluid ext. valerian, where there were aggravating and seemingly useless pains at the commencement of labor, with good effects.

In one case of asthma, I gave the salt, but its use has not been followed up closely, as the patient is well-nigh cured since leaving the Western Reserve, without any medicine.

As a calmative and hypnotic, it has been used in excessive wakefulness and nervous irritability for some time, and I have frequently given it to adults to cause sleep.

Respecting its *modus operandi* I have but little to say, except that it seems most efficient in cases where *mucous* surfaces are affected, and consequently morbidly sensitive. It acted thus in disease of the mucous coat of the bladder, and the alimentary canal. If it relieves asthma, it will be more effective in that variety caused by the action of fog, and smoke, fumes of various kinds, ipecac, or hay, etc., than in cases of a reflex origin, or of those depending upon disease of the heart, or other thoracic organs.

